# Nanopore Sequencing Reveals Novel Alternative Splice Variants of EZH2 in Pediatric Medulloblastoma

**DOI:** 10.3390/biomedicines13102461

**Published:** 2025-10-10

**Authors:** Josselen Carina Ramírez-Chiquito, Sergio Antony Rosete-Ambriz, Ana Consuelo Olguín-García, María del Pilar Eguía-Aguilar, Ana Maria Niembro-Zuñiga, Alfonso Marhx-Bracho, Mario Perezpeña-Diazconti, Sergio Juárez-Méndez

**Affiliations:** 1 Experimental Oncology Laboratory, National Institute of Pediatrics, Mexico City 04530, Mexico; carina-rc@outlook.com (J.C.R.-C.); sergioantonyr@gmail.com (S.A.R.-A.); ana_hjg@hotmail.com (A.C.O.-G.); 2 Postgraduate in Biological Sciences, Postgraduate Unit, Building D, 1st Floor, Postgraduate Circuit, University City, Coyoacan, Mexico City 04510, Mexico; 3 Experimental Pathology Research Laboratory, Children’s Hospital of Mexico, Federico Gómez, Mexico City 06720, Mexico; eguiapilar@yahoo.com.mx; 4 Department of Clinical and Experimental Pathology, Children’s Hospital of Mexico, Federico Gómez, Mexico City 06720, Mexico; 5 Department of Pediatric Oncology, National Institute of Pediatrics, Mexico City 04530, Mexico; ananiembro@hotmail.com; 6 Department of Surgery, National Institute of Pediatrics, Mexico City 04530, Mexico; marhxalfons@yahoo.com.mx; 7 Molecular Pathology Laboratory, Department of Pathology, National Institute of Pediatrics, Mexico City 04530, Mexico; mpdiazconti@gmail.com; 8 Department of Pathology, National Institute of Pediatrics, Mexico City 04530, Mexico

**Keywords:** ONT, transcriptome, alternative splicing, RNA isoforms, medulloblastoma

## Abstract

**Background:** Medulloblastoma is the childhood tumor with the highest morbidity and mortality worldwide. This type of cancer is characterized by a high degree of heterogeneity that gives rise to different molecular groups with disparities in the clinical presentation and prognosis. Among the molecular differences, one of the most relevant factors is alternative splicing, as it is responsible for transcriptomic diversity. *EZH2* is a gene processed by alternative splicing that functions as an epigenetic regulator. In cancer, certain *EZH2* mRNA variants are associated with tumorigenesis; however, in medulloblastoma, the alternative splicing pattern of *EZH2* has not been studied. Currently, the best tool for identifying alternative splicing variants is long-read sequencing. **Methods:** We amplified the most variable region of *EZH2* alternative splicing and used nanopore sequencing to obtain the transcriptional profile of the gene in patients with medulloblastoma. We verified the variants identified with Sanger sequencing and digital RT–PCR. Finally, we studied the relationship between the expression levels and the clinical–biological characteristics of the patients. **Results:** We identified seven mRNA variants of *EZH2* expressed in medulloblastoma patients, five of which had not been reported previously. In addition, high expression of the novel variant *EZH2*_RetI8 was associated with patient mortality (*p* < 0.05). **Conclusions**: This is the first evidence of the *EZH2* mRNA variant profile in medulloblastoma, revealing seven alternative transcripts, one of which is associated with patient mortality. This is a clear example of the complexity of the transcriptome and how long-read sequencing can resolve alternative splicing patterns.

## 1. Introduction

Medulloblastoma (MB) is the most common solid malignant neoplasm in the pediatric population worldwide, with two peaks of incidence, at 4 and 8 years of age, as well as a male predominance with a 3:2 ratio. A high percentage of patients are diagnosed in the metastatic stage, and approximately 40% of patients experience recurrence and death; consequently, overall survival at five years is between 50% and 85% [1,2,3]. This type of tumor is characterized by high biological and clinical heterogeneity; therefore, its classification has evolved from histopathological to molecular characteristics [3,4].

The most important factor for prognosis is the molecular classification: WNT-activated, SHH-activated, group 3, and group 4 [5]. WNT tumors represent only 10% of the total cases and constitute the group with the best survival rate. The SHH group comprises 30% of patients with intermediate prognoses, whereas 25% of patients with medulloblastoma are in Group 3, the group with the worst prognosis. Finally, Group 4 is the most common type, accounting for approximately 35% of cases with an intermediate prognosis [6,7,8]. However, Williamson et al. demonstrated the presence of mixed or intermediate tumors, which present combined characteristics between the molecular groups [9]. These characteristics, combined, represent another important challenge for diagnostic centers, where experience in the molecular classification of MB is limited.

The WHO Classification of Tumors of the Central Nervous System (CNS), published in 2021, established a combination of histological and molecular features for classifying medulloblastoma [4]. Currently, reference methods for molecular classification are based on gene expression profiles and DNA methylation patterns, both of which are regulated by various processes, such as alternative splicing and epigenetic regulators, respectively. Interestingly, in this tumor type, regions with a high prevalence of hypomethylation were identified to be correlated with increased gene expression [10].

In this context, one of the most important genes is the Enhancer of Zeste Homolog 2 (*EZH2*), the catalytic subunit of Polycomb repressor complex 2 (PRC2), a key epigenetic regulator of cellular differentiation and developmental programs. PRC2 facilitates gene silencing primarily by modulating chromatin structure, where EZH2 performs the trimethylation of Lys27 at histone 3 (H3K27me3) [11]. In addition, EZH2 can methylate nonhistone targets and interact with other proteins, regulating the cell cycle in a PRC2-independent way [12,13,14].

Dysregulation of EZH2 has been associated with the development and progression of many types of cancer [15,16,17]. In medulloblastoma, EZH2 plays dual roles as a tumor suppressor and oncogene, depending on the target genes it regulates, and has been shown to be involved in the regulation of stemness features in medulloblastoma cells [18,19,20]. Among the alterations that affect the expression and catalytic function of EZH2 in cancer, translocations, gene amplifications, mutations in functional domains and alternative transcripts have been described [21,22,23,24,25,26,27].

Alternative splicing (AS) is a key process in the diversity of the transcriptome/proteome and is estimated to occur in more than 85% of human genes, with an average of seven transcripts per gene [28,29]. In the case of *EZH2*, 20 different transcripts have been identified, six of which encode five isoforms, and the rest have been described as noncoding RNAs. *EZH2-201* is considered the canonical transcript and consists of 20 exons and encodes the longest isoform, whereas the other five transcripts are characterized by splice regions in frame exons or 5′-UTRs. In liver and kidney malignancies, alternative *EZH2* variants have been shown to contribute to tumorigenic potential [25,26]; however, in medulloblastoma, which *EZH2* variants are expressed and what their potential impact on the disease has not been studied.

A valuable tool for detecting splice variants is long-read sequencing (LRS), a technology capable of generating sequence reads of thousands of bases in length, which is different from short-read sequencing, which generates reads with a length limit of base pairs. Long reads allow the spanning of large regions with the potential to recover high numbers of transcripts, in contrast to short-read sequencing, which is limited to shorter regions and may miss certain splicing events. Oxford Nanopore Technologies (ONT), one of the main LRS technologies, is based on real-time detection of nucleotides passing through a protein nanopore by measuring the ionic current.

In this work, we used ONT sequencing to determine alternative *EZH2* variants expressed in tumor samples from medulloblastoma patients and analyzed the region that presented the greatest number of splicing events and covered exons 9 to 13 [27]. Additionally, we analyzed associations between expression levels and clinical–biological features.

## 2. Materials and Methods

### 2.1. Biological Material

For this study, samples from primary tumors of pediatric patients with medulloblastoma were obtained at the time of the initial operation. None of the patients had received any therapy prior to surgery, and all diagnoses were confirmed by a pathologist via histological evaluation of the tumor specimen. Molecular classification was performed via microarray analysis.

All procedures were carried out after the patients signed the informed consent form, which was approved by the Institutional Ethics Committee (protocol INP 2022/003) CONBIOÉTICA-09-CEI-025-20161215 in accordance with the Declaration of Helsinki. The collected samples were subsequently stored in RNAlater (Qiagen, Hilden, Germany) at −70 °C until use for RNA purification.

### 2.2. RNA Purification

Fresh tumor samples were disrupted via a TissueLyser system (Qiagen, Hilden, Germany) for 60 s at 25 Hz. Total RNA was extracted via an RNeasy Mini Kit (Qiagen, Hilden, Germany) according to the manufacturer’s protocol and previous reports [30]. The concentration was determined with a NanoDrop One UV–Vis Spectrophotometer (Thermo Fisher Scientific, Waltham, MA, USA), and total RNA was stored at −70 °C until use for cDNA synthesis.

### 2.3. cDNA Synthesis

Before cDNA synthesis, to avoid DNA contamination in the samples, 1 µg of total RNA was treated with 1 U of DNase (Thermo Fisher Scientific, Waltham, MA, USA) and incubated at 37 °C for 30 min. Then, 1 µL of 50 mM EDTA was added to stop the reaction at 65 °C for 10 min.

cDNA synthesis was performed via a Revert Aid Transcriptase Kit (Thermo Fisher Scientific, Waltham, MA, USA); the reactions contained the following components: 5× RT buffer, 1 mM dNTP mixture, random hexamers (100 pmol), RiboLock RNase Inhibitor (10 U) and RevertAid Reverse Transcriptase (200 U). The reaction mixture was incubated for 10 min at 25 °C, 60 min at 60 °C, and 10 min at 70 °C.

### 2.4. RT–PCR

For nanopore sequencing, we amplified a region of *EZH2* that spans from exon 7 to exon 14 with the following primers: forward, 5′-GATAAGGGCACAGCAGAAGAA-3′ and reverse, 5′-GTGGATGATCACAGGGTTGATAG-3′, as part of a sequencing panel that included 14 other genes. For the PCRs, 25 ng of cDNA from patients with medulloblastoma was added to the PCR mixture, with 10 µM primer forward and reverse, 15 µL of Kapa 2G Fast HotStart Ready Mix (Kapa Biosystems, Wilmington, MA, USA) and free nuclease water up to a final volume of 30 µL. The temperature cycle was as follows: predenaturation to 95 °C for 1 min, followed by 35 cycles of 95 °C for 15 s, 55 °C for 15 s, and 72 °C for 30 s, with a final extension of 72 °C for 5 min. Amplicons were confirmed via agarose gel electrophoresis (1.5%) and then purified via ethanol precipitation.

### 2.5. PCR Product Purification

The PCR product was first mixed with an equivalent volume of water and 600 μL of cold isopropanol, followed by precipitation in ice (−20 °C) for 30 min. Then, the mixture was centrifuged at 14,000 rpm for 10 min to discard the supernatant, followed by two washes as follows: (1) 600 μL of cold 70% ethanol and (2) centrifugation at 14,000 rpm for 5 min. The supernatant was discarded, and the mixture was left upside-down for 1 h until the ethanol evaporated. Afterwards, the pellet was eluted in nuclease-free water and quantified with a Qubit DNA HS Assay Kit (Thermo Fisher Scientific, Waltham, MA, USA) according to the manufacturer’s protocol.

### 2.6. Library Preparation and Nanopore Sequencing

The library with multiplex samples and prepared purified PCR products for long-read sequencing was generated via a Native Barcoding Kit 24 V14 (SQK-NBD114.24) from Oxford Nanopore Technologies (Oxford Nanopore Technologies, Oxford, UK). First, 200 fmol of each amplicon end was prepared for adapter attachment with the NEBNext Ultra II End repair/dA tailing module (New England Biolabs Inc., Ipswich, MA, USA).

Next, the unique native barcodes were ligated onto the end-prepared cDNA of each sample with NEB Blunt/TA Ligase Master Mix (New England Biolabs Inc., Ipswich, MA, USA). All the samples were subsequently mixed, and the sequencing adapters were ligated with the NEBNext Quick Ligation Reaction Module (New England Biolabs Inc., Ipswich, MA, USA).

Fifty fmoles of the library were loaded into a FLO-MIN114 flow cell with R10.4.1 chemistry following the manufacturer’s instructions. The sequencing was performed in the MinION Mk1C device (Oxford Nanopore Technologies, Oxford, UK), with the following parameters in MinKNOW (version 24.06.5) software: pore scan frequency of 1.5 h, basecalling with Dorado v7.4.14 software using the fast model v4.3.0, minimum Q score filtered 8, barcode timing and mid-read barcode filtering off. The run time was 5 h.

### 2.7. Bioinformatic Analysis of Long-Read Sequencing Data

Basecalling was performed in real time during the sequencing run, with Dorado v7.4.14 software via the fast model (version 4.3.0) and a Q score filter of 8. The results were classified into passed reads or failed reads and separated per barcode. The passed reads were used to perform a second basecalling with Dorado v7.4.14 software and the high-accuracy model, which considers a Q threshold of 9 and a trimming of adapter sequences. Those read files were used for the downstream analyses.

The FASTQ files containing the raw sequencing data were aligned against the human reference genome (GRCh38) via the minimap2 method (version 2.28) in Galaxy (version 2.2.1+galaxy1) online software (https://usegalaxy.org/, accessed on 24 September 2025) [31,32]. BAM and alignment indexed (.bai) output files, obtained for each sample with alignments against the reference genome, were used for data visualization and Sashimi plot creation via Integrative Genomics Viewer (IGV) (version 2.18.4) software, considering only the alignments in the reverse strand according to the localization of EZH2 and a minimum coverage junction of 8.

The coverage of each transcript was obtained via the StringTie (version 2.2.3) method [33,34], and the sequences were extracted with the gffread tool (version 0.12.7) from the sorted BAM file in Galaxy. To identify regions of alternative splicing in the assembled transcripts, we manually compared the sequences with the canonical *EZH2* transcript (ENST00000320356.7). To determine whether the transcripts were novel or previously reported variants, we subsequently performed an alignment against the *EZH2* cDNA sequences contained in Ensembl via the online tool Clustal Omega (https://www.ebi.ac.uk/jdispatcher/msa/clustalo?stype=dna, accessed on 22 February 2025) [35]. Finally, transcript counts (transcripts per million, TPM) were used to graphically represent the abundance of each variant per sample and create a clustering heatmap in the online software SRPlot (https://www.bioinformatics.com.cn/srplot, accessed on 13 April 2025) [36].

### 2.8. Sanger Sequencing

To validate the RetI8, RetI9 and OmE11 transcripts, we used the same primers used in the long read sequencing (forward, 5′-GATAAGGGCACAGCAGAAGAA-3′ and reverse, 5′-GTGGATGATCACAGGGTTGATAG-3′), following the same conditions as those used in the PCRs for nanopore sequencing: predenaturation to 95 °C for 1 min, followed by 35 cycles of 95 °C for 15 s, 55 °C for 15 s, and 72 °C for 30 s, with a final extension of 72 °C for 5 min.

The PCR products were separated via electrophoresis (2.5% agarose), and the DNA was purified via a QIAquick Gel Extraction Kit (Qiagen, Valencia, CA, USA). The amplicons were subsequently subjected to Sanger sequencing via the ABI Prism 3130 Genetic Analyzer (Applied Biosystems, Foster City, CA, USA) with the BigDye Terminator v3.1 cycle sequencing Kit (Applied Biosystems) according to previous reports [37]. The electropherograms were visualized with BioEdit (version 7.7.1) software, and the sequences obtained were aligned against the reference sequence annotated at the National Center for Biotechnology Information (NCBI).

### 2.9. RNAseq Data Mining and Bioinformatic Analysis

Data mining was performed in the Gene Expression Omnibus (GEO) database, with the searches filtered for RNA-seq data from pediatric patients with medulloblastoma. These data were obtained from six patients with group 4 medulloblastoma and three patients with group 3 medulloblastoma via the Illumina NovaSeq 6000 platform (GEO accession number: GSE243682). Bioinformatic analysis was performed on the Galaxy platform v2.2.1+galaxy1. Initial quality control was carried out via fastqc (version 0.12.1), and adapter sequences that were removed via the fastp (version 0.23.2) tool were identified. Sequence alignment was performed via RNA STAR (version 2.7.11a) [38] against the GRCh38 reference genome and the GENCODE v47 reference transcriptome, employing the parameters recommended by Gallardo et al. [39]. Transcript assembly and quantification were conducted via StringTie (version 2.2.3) [33,34], and the resulting sequences were extracted via gffread (version 0.12.7). The sequences corresponding to *EZH2* were subsequently identified and manually compared to the canonical transcript (ENST00000320356.7) to detect alternative splicing patterns.

### 2.10. Digital PCR

To verify the results of the NGS data analysis, we designed specific PCR primers for *EZH2_RetI8* and *EZH2_RetI9* via the Primer-BLAST online tool v1.0.1 (https://www.ncbi.nlm.nih.gov/tools/primer-blast/, accessed on 13 March 2025) (Table 1).

Digital PCR (dPCR) was performed via the QIAcuity One system (Qiagen, Hilden, Germany). The reaction mixture was prepared according to the manufacturer’s instructions, with the following composition: 4.0 μL of QIAcuity EG Master Mix 3× (Qiagen, Hilden, NW, DE), 1 µM of each primer (forward and reverse), and 25 ng of cDNA template, resulting in a final reaction volume of 12 μL.

The prepared reaction mixtures were transferred to a QIAcuity 8.5k 96-well nanoplate for partitioning via the standard priming protocol provided by Qiagen. The cycling conditions consisted of initial enzyme activation at 95 °C for 2 min, followed by 40 cycles of denaturation at 95 °C for 15 s, annealing at 60 °C for 15 s, extension at 60 °C for 15 s, and a final step at 40 °C for 5 min. Partitioning was achieved with a standard exposure time on the green channel.

Data analysis was performed via QIAcuity Suite (version 2.0.20) software, and concentrations were exported as copies/μL. For statistical analysis, first, we calculated the concentrations for copies/sample considering the concentration and volume used in the experiments; then, we normalized the values of *EZH2_RetI8* and *EZH2_RetI9* expression to those of the reference gene *PGGT1B* [30] (Table 1). For statistical comparisons, the type of distribution of each dataset was defined by the Kolmogorov–Smirnov test (*p* < 0.05), and multiple or pair comparisons with nonparametric tests (Kruskal–Wallis or Mann–Whitney tests, *p* < 0.05) were conducted with GraphPad Prism (version 10.4.1) software.

## 3. Results

### 3.1. Patient Cohort

A total of 23 pediatric patients with medulloblastoma were included in the study. The epidemiological characteristics are summarized in Table 2, where we can observe a male predominance (2:1) and a peak incidence at 8 years, as has been described in other populations [3,40,41,42]. Interestingly, the distribution of molecular groups differed from that reported in most studies, since the most frequent group was SHH, not Group 4 [5,41,43,44].

### 3.2. Nanopore Sequencing Reveals New Junction Patterns in EZH2 Transcripts Expressed in Childhood Medulloblastoma

**Figure 1:**
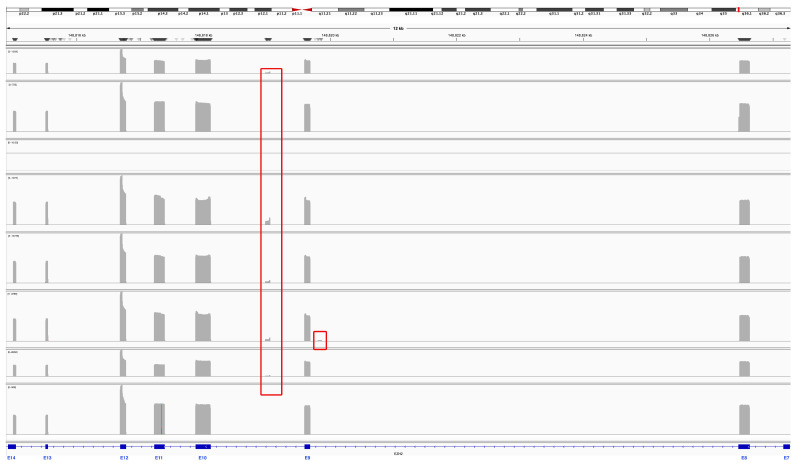
**Coverage track of the alignment to the *EZH2* reference sequence.** The reads aligned against the *EZH2* reference sequence for each sample are presented in each row. In gray, spikes in coverage are observed at exonic sequences of *EZH2*, and red rectangles indicate reads in introns 8 and 9.

**Figure 2:**
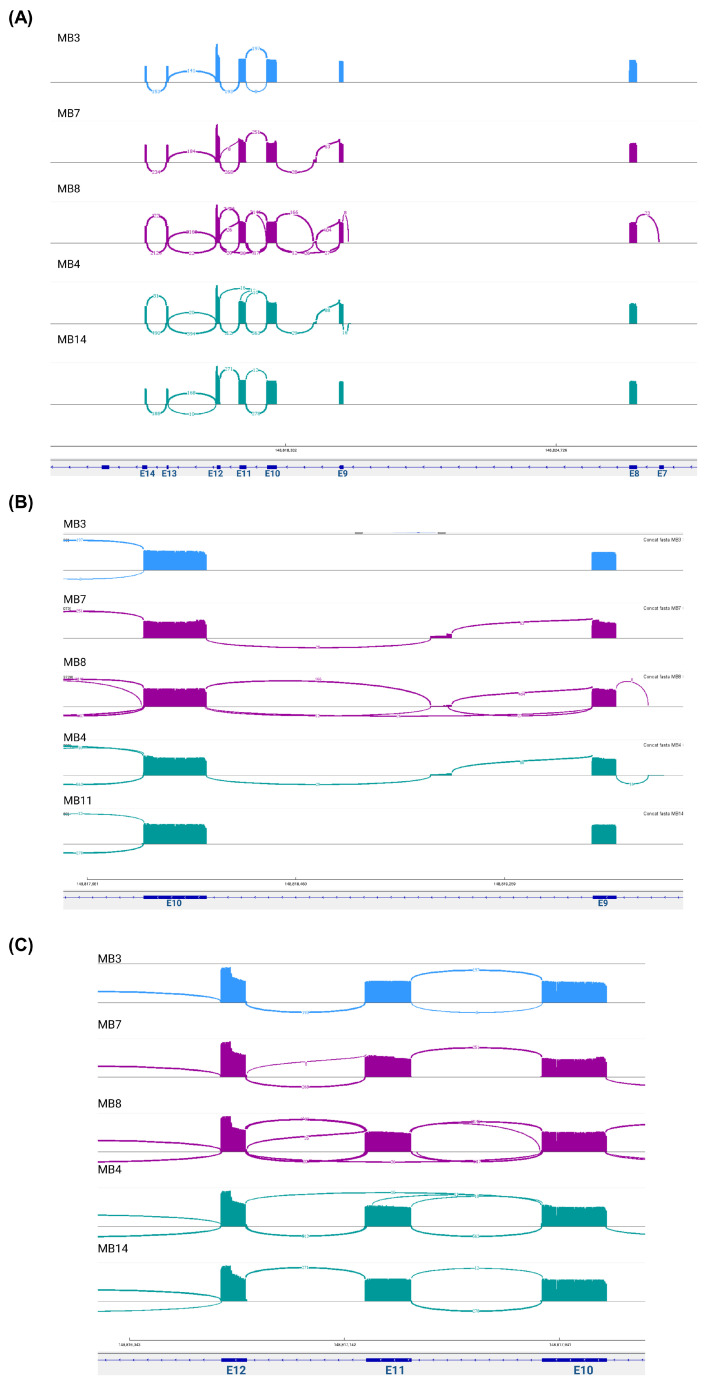
**Novel splice junction patterns in *EZH2* transcripts expressed in medulloblastoma.** The Sashimi plots visualize the read coverage and junction reads, which are plotted as arcs with the number of splicing counts. (**A**) The binding patterns along the sequenced region, (**B**) including partial retention of intron 9 and intron 8 and (**C**) excluding exon 11. The colors represent the molecular group: blue-SHH; purple-group 3; and green-group 4.

Eight samples from pediatric patients with medulloblastoma (Supplementary Table S1) were subjected to *EZH2* RT–PCR amplification with primers spanning exons 7 to 14 (Figure 1 and Figure 2A). Two of these samples were SHH, three were G3, and three were G4. The amplicons of *EZH2* were sequenced in a multiplex assay with the Oxford Nanopore platform, and the resulting reads of each sample were processed to visualize the alignment reads and the transcripts ensembled with Sashimi plots; moreover, the sequences were extracted to verify those junctions (Supplementary File S1). We obtained reads annotated to *EZH2* in all samples except MB5, probably because of low sequencing depth for this sample or because MB5 did not express *EZH2* or its expression was very low. For the remaining samples, alignments were visualized with IGV, and interestingly, we identified alignment reads in intronic regions of approximately 80 bp (Figure 1).

To confirm reads in the intronic regions, Sashimi plots were constructed. Unfortunately, for two samples (MB1 and MB11), we did not visualize these graphs, possibly due to the low number of aligned reads obtained from these patients. However, alternative *EZH2* transcripts were identified in the other samples. Multiple junction patterns between reads in exonic and intronic regions were observed (Figure 2A). Interestingly, in some samples, we identified the junction between exon 9 and reads in a region of intron 9 and the junction between exon 8 and reads in a region of intron 8 (Figure 2B), and in two samples (MB4 and MB8), the junctions between exon 10 and exon 12 were visualized, suggesting the omission of exon 11 (Figure 2C).

To corroborate these findings, we extracted the assembled transcripts and compared them with the canonical transcript sequence obtained from Ensembl. As expected, in all samples except for sample MB4, we obtained the canonical transcript that included all complete exons covered by the primers (E7–E14), named *EZH2-V1*. Moreover, we verified the transcripts identified with the Sashimi plots, with the omission of exon 11 and the transcripts with partial retention of intron 9 with a size of 83 bp (RetI9) and partial retention of intron 8 with a size of 62 bp (RetI8) (Supplementary File S1).

**Figure 3:**
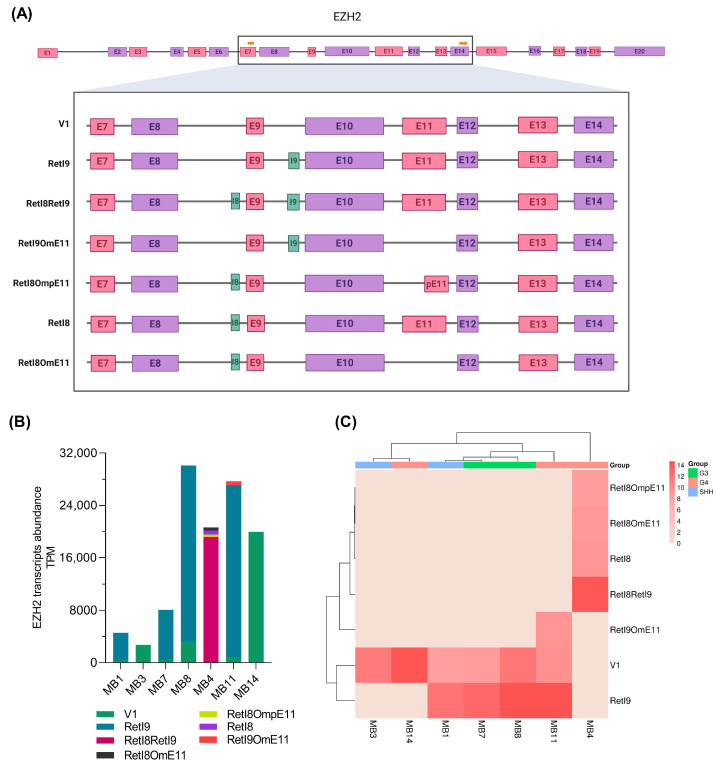
***EZH2* transcriptomic patterns in patients with medulloblastoma**. (**A**) The scheme summarizes the alternative *EZH2* transcripts that were revealed in the samples from patients with medulloblastoma; the first two variants are those previously reported, whereas the last five correspond to the newly elucidated variants. At the top, the orange lines represent the locations of the primers used for *EZH2* amplification, and the black rectangle indicates the sequenced region. At the bottom, the green regions represent intronic retention, whereas the pink and purple rectangles represent exons. (**B**) The bar graph shows the abundance of *EZH2* transcripts obtained from nanopore sequencing for each sample in transcripts per million (TPM). (**C**) Hierarchical clustering heatmap constructed from the abundance data; the molecular group of each patient is shown.

We identified other mRNA variants resulting from the combination of these alternative regions: the transcript with both retention of introns 8 and 9 (RetI8RetI9), the transcript with retention of intron 8 and omission of exon 11 (RetI8OmE11), the transcript with retention of intron 9 and omission of exon 11 (RetI9OmE11) and the transcript with retention of intron 8 and partial omission of exon 11 (RetI8OmpE11). All these combinations of alternative mRNA expression data for *EZH2* are presented in Figure 3A, and the complete sequences are included in Supplementary File S1.

### 3.3. Expression Profile of Alternative EZH2 Transcripts

To generate the *EZH2* variant profile for each sample, we obtained the abundance of the transcripts in transcripts per million (TPM) and constructed a bar graph (Figure 3B). In two patients (MB3 and MB14), only the canonical *EZH2-V1* variant was revealed; in the other patients (MB1, MB7 and MB8), two transcripts were detected; and in samples MB11 and MB4, three and four *EZH2* variants were detected, respectively. Next, to analyze the association between the *EZH2* alternative transcript profile and the molecular group, we performed a clustering method (Figure 3C), which revealed that a similar expression pattern allowed clustering of Group 3 samples, whereas for Group 4 samples, the expression patterns were more diverse, placing one sample within the SHH samples.

### 3.4. Validation of New EZH2 Transcripts

**Figure 4:**
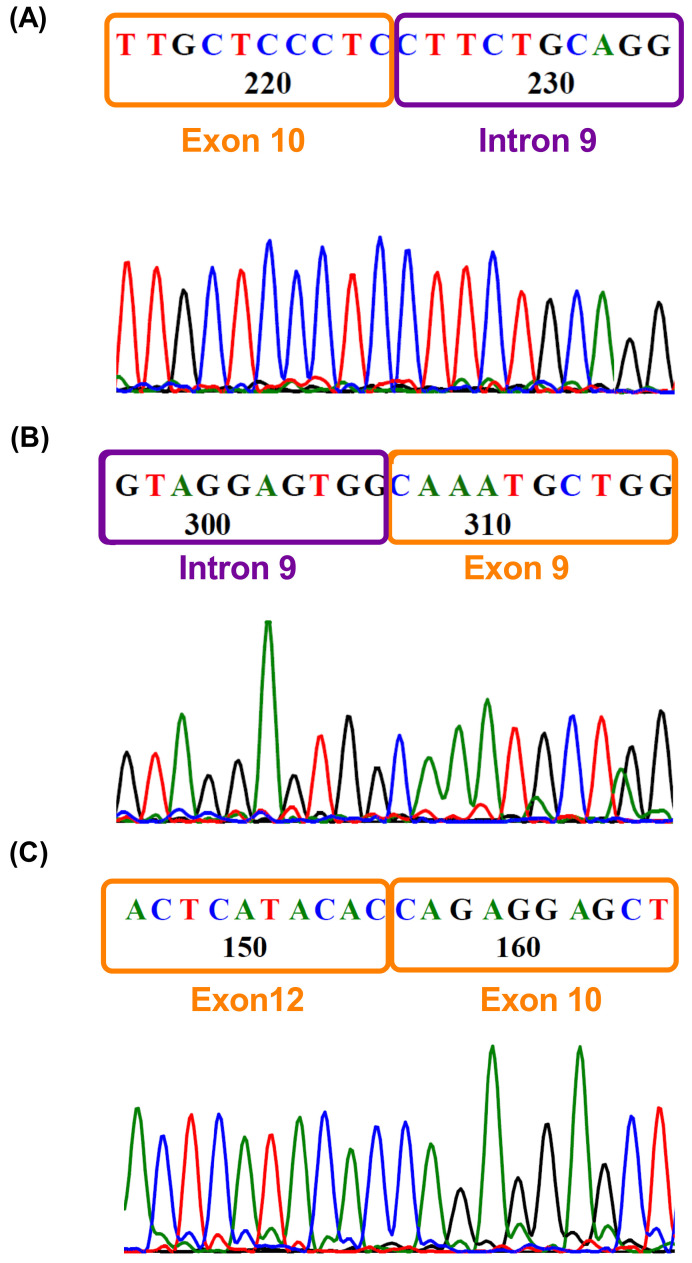
**Validation of variants by Sanger sequencing**. Electropherograms obtained by Sanger sequencing with reverse primers showing (**A**) the junction of exon 10 and intron 9 and (**B**) the junction of intron 9 and exon 9, corresponding to the RetI9 variant. (**C**) Junction between exons 10 and 12, depicting the omission of exon 11.

To validate the RetI8, RetI9 and OmE11 transcripts, we used the same primers as those used for long-read sequencing and performed Sanger sequencing (Figure 4). We confirmed two alternative transcripts: the partial retention of intron 9 (Figure 4A,B) and the omission of exon 11 (Figure 4C). The retention of intron 8 was not identified due to difficulty in purifying the PCR products since the differences in bp between the variants are very small. In addition, the 5′ and 3′ ends in the sequences were contaminated with other sequences, so we could not define a sequence. Additionally, to confirm these findings, we performed gene expression data mining in patients with medulloblastoma via short-read sequencing with an Illumina platform. Our findings revealed the presence of a splicing variant that includes RetI9 (Supplementary File S1). Unfortunately, owing to the type of sequencing, it is very difficult to demonstrate the other patterns of splicing observed by long-read sequencing (Supplementary Figure S1). In addition, expression levels play a crucial role in identifying variants whose expression is lower than that of canonical variants. Although the results from short-read sequencing are limited, we were able to confirm the presence of at least one retention site (RetI9).

### 3.5. Quantification of the Novel EZH2 Transcripts RetI8 and RetI9 in Patients with Medulloblastoma

Finally, we performed absolute quantification of the new transcripts that included a single splice region (RetI8 and RetI9), as the remaining variants included more than one alternative splicing region, such as RetI8OmE11 or RetI8RetI9, which cover more than 500 bp and, for this reason, could not be quantified by digital PCR.

**Figure 5:**
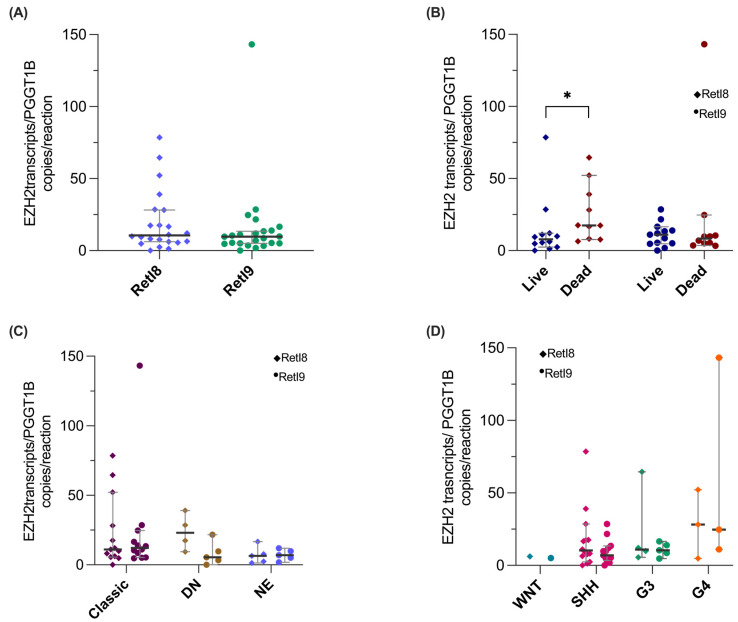
**Validation of *EZH2* RetI8 and RetI9 transcripts via digital PCR.** The graphs show the expression normalized to the reference gene *PGGT1B* of the RetI8 (rhombuses) and RetI9 (circles) variants in (**A**) patients with medulloblastoma (N = 23) and in patients classified on the basis of their (**B**) mortality, (**C**) histologic type and (**D**) molecular group. For all the graphs, the median and 95% CI data are shown, and the groups were compared with nonparametric tests, which revealed a statistically significant difference between high RetI8 expression and mortality (* = *p* < 0.05). DN: Nodular Desmoplastic, EN: Extensive Nodularity.

We found that the RetI8 and RetI9 transcripts were expressed at similar levels (Figure 5A). Next, we analyzed the associations between the expression levels and the clinical or molecular characteristics of patients. For survival status, the RetI8 variant was overexpressed in deceased patients, whereas for histopathological classification (Figure 5C) and molecular groups (Figure 5D), the expression of the two alternative EZH2 variants was similar between groups.

## 4. Discussion

Medulloblastoma is the most common SNC childhood cancer and, unfortunately, has a low survival rate [6,45,46]. This malignant neoplasm is characterized by high intra- and intertumor heterogeneity; therefore, the most successful treatment strategy relies on molecular classification [47]. On the basis of gene expression profiles, medulloblastomas are classified into four groups: WNT, SHH, Group 3 and Group 4. Recently, with the addition of DNA methylation patterns, twelve subgroups were identified (two WNT, four SHH, and three subgroups for G3 and G4). However, the most important prognostic factor remains classification at the group level [3,5,47].

Differences at the molecular level include mutations, amplifications, and gene expression and DNA methylation profiles [48,49,50]. Among the mechanisms involved in regulating the gene expression landscape, alternative splicing is a key process that diversifies the transcriptome and proteome. Similarly, the modulation of DNA methylation via epigenetic regulators is another mechanism that generates different gene expression patterns. One gene that is regulated by alternative splicing, which in turn acts as an epigenetic regulator, is *EZH2*, which encodes the enzimatically active subunit of polycomb repressive complex 2, a catalyst of histone H3 methylation. EZH2 is relevant in normal processes such as cellular differentiation and development, but its deregulation has been associated with multiple hallmarks of cancer, such as stemness, proliferation, apoptosis and drug resistance.

In medulloblastoma, *EZH2* is highly expressed and is associated with poor prognosis [51,52,53]. However, in some studies, *EZH2* has been shown to have dual functions. In 2012, Smith and colleagues reported that EZH2 enhances resistance to apoptosis through the negative regulation of *DAB2IP* [18]; conversely, a study in a murine model revealed that the repression of *EZH2* accelerated tumorigenesis [19], and in another investigation with a stem cell model, it was demonstrated that *EZH2* is necessary to maintain the characteristics of stemness [20].

Although the involvement of *EZH2* in the development and progression of medulloblastoma is relevant, few alterations that modify its function have been described; mutations in the functional domains that prevent methylation catalysis have been reported in other types of cancer [21,22,23,54,55,56], but its posttranscriptional regulation in medulloblastoma has not been studied. In this work, we amplified and sequenced the most variable alternative splicing region of *EZH2* [27], revealing the *EZH2* transcriptional profile in pediatric patients with medulloblastoma. We identified two previously reported mRNA variants: the canonical transcript that includes all covered exons in the sequenced region (*EZH2-V1*) and the variant RetI9, which includes a region of intron 9 (corresponding to the ENST00000682263.1 transcript). Moreover, we discovered five novel mRNA variants expressed in medulloblastoma patients, characterized by partial retention of introns 8 and/or 9 combined with total or partial omission of exon 11 (Figure 1, Figure 2 and Figure 3A).

The canonical variant of *EZH2* consists of 7 domains: the WD-binding domain (encoded by exon 3) is located in the N-terminal region, which interacts with EED (embryonic ectoderm development), another component of the PCR2 complex, followed by domain I, which is the PHF1 binding region, and domain II or MCSS (encoded by exons 8 and 9), which is involved in the junction with SUZ12; two SANT domains, SANT1, which is encoded by exons 5 to 8; and SANT2 (encoded by exons 11 and 12), which are both responsible for interaction with histones; and in the C-terminal region, the CXC domain (cystein-rich domain), which is encoded by exons 16 and is necessary for binding other PRC2 regulatory proteins; and the SET domain (enhancer of zeste and trithorax domain), which encodes from exons 17 to 20 and is responsible for methyltransferase activity [26,27,57,58,59].

On the basis of the transcript structure of *EZH2*, the novel variants that include the retention of introns 8 and/or 9 could have modifications in the MCSS domain, altering the correct assembly of the PRC2 complex, and consequently could be reflected in the deregulation of gene repression, which, depending on the genes involved, could translate into an increase in tumorigenic potential. Furthermore, it has been reported in chronic myeloid leukemia that the inclusion of a sequence between exons 8 and 9 of *EZH2* gives rise to a premature stop codon with prediction of nonse-mediated decay (NMD) [60]; therefore, we cannot exclude the possibility that this same process occurs with the transcripts evidenced here. However, functional analysis is necessary to test these assumptions.

Similarly, in acute myeloid leukemia, the highest number of splicing events in *EZH2* occurs between exons 9 and 13 [27]. By including this region in the sequencing data (exons 7 to 14), our findings were consistent with the excision of exon 11 (Figure 3A). Moreover, we were able to identify combinations of splicing events that have not been previously reported. For example, we found *EZH2* variants with total or partial skipping of exon 11, which also included retention of intronic regions (intro 8 or intron 9), and we believe that these splicing patterns could generate isoforms with loss or decrease of two of their key functions: 1) catalysis of H3K27me3, as a consequence of probable structural changes in the PCR2 factor binding domains (PHF1 and SUZ12), and 2) chromatin remodeling and transcriptional modulation, owing to the loss of the coding region (exon 11) of the second SANT domain required for DNA binding [61,62].

One of the limitations of this study is that we covered only one region (exons 7 to 14) of the complete transcript; therefore, other alternative splicing patterns involving the unsequenced regions were not studied. However, it has already been reported that the regions with the greatest number of splicing events occur in exons 9–14 [27], and via long-read sequencing, we were able to identify binding regions involving new alternative splice sites and their combinations, i.e., variants that have more than one alternative splice site.

Researchers have elucidated alternative *EZH2* variants and their associations with tumorigenesis and disease prognosis. In hepatocellular carcinoma (HCC), the *EZH2* variant that excludes exon 4 is associated with poor survival, whereas the omission of exon 14 (EZH2∆14) gives rise to an isoform with antagonistic functions to the canonical protein, inhibiting H3K27me3 methylation and increasing the stability of target regions in the mRNA, in addition to being positively correlated with patient survival [26,27]. Similarly, in renal cancer, *EZH2∆14* has lower tumorigenic potential than the canonical variant [25]. In chronic myeloid leukemia, the *EZH2* variant lacking exons 11 and/or 12 is related to decreased protein expression of EZH2; as a possible explanation, Shiozawa et al. reported that the skipping of exon 11 leads to a premature stop codon [63].

Herein, we found that the novel alternative variant RetI8 was overexpressed in deceased patients with medulloblastoma, and we found no association between RetI8 or RetI9 expression and the histologic or molecular group (Figure 5C,D), which may be attributed to the low number of patients included in the analysis. However, when we associated the complete profile of the alternative *EZH2* transcripts with the molecular group, we observed some degree of concordance, since only one patient in group 4 was integrated among the profiles of the SHH patients, whereas the rest of the profiles corresponded with the molecular group (Figure 3C). Although molecular typing is one of the most important prognostic factors in medulloblastoma, discrepancies in molecular classification have been observed, which is due to high tumor heterogeneity. However, our findings revealed an association of the *EZH2* RetI8 variant with deceased patients, which led us to believe that this variant could play an important role in the biology of medulloblastoma, although further studies will be necessary.

Most samples expressed between one and two *EZH2* transcripts (Figure 3B), including the canonical variant and the variant with intron 9 retention; however, one patient with group 4 medulloblastoma expressed four alternative *EZH2* transcripts and had no expression of the canonical variant, so we speculate that one of these alternative variants could negatively regulate the canonical transcript, as has been observed with the variant lacking the SET domain in a group 3 medulloblastoma model [19].

In the last decade, different EZH2 inhibitors have been designed and included in preclinical and clinical trials. In medulloblastoma, most studies have indicated that tazemetostat decreases tumor proliferation and improves survival; however, in another study, tazemetostat had no antitumor effect on medulloblastoma [52,64,65]. Resistance to the drug could be related to the *EZH2* transcript profile expressed by each patient, since the structural variation produced by the alternative variants of the gene could intervene with drug binding in the SET domain, which would decrease the therapeutic effect, as has been observed with other genes, whose alternative splice variants are associated with resistance to treatment [66,67,68,69].

## 5. Conclusions

Taken together, these results provide the first evidence of the *EZH2* mRNA variant profile in medulloblastoma, revealing the wide landscape of its expression in this disease. Seven transcripts were identified, five of which had not been reported previously, representing a clear example of the complexity of the transcriptome and how long-read sequencing allows alternative splicing patterns to be resolved. In addition, the novel alternative variant RetI8 was shown to be associated with mortality in patients with medulloblastoma.

Although the current work revealed *EZH2* transcriptional profiles, further studies must be conducted to elucidate the biological functions of all novel alternative transcript variants and potential isoforms.

## Figures and Tables

**Table 1:** List of primers used for the quantification of EZH2 transcripts.

Gene	Sequence (5′->3′)	Tm (°C)	Product Size (bp)
**EZH2_RetI8**	Fw CTAGAGCTGTTTCTGTGTTCT Rv CAGTAAGAGCCTGAAGGAAAG	60 60	84
**EZH2_RetI9**	Fw CCAGCATTTGCCACTCCTACCT Rv TTTGCTCCCTCCTTCTGCAGGT	60 60	103
**PGGT1B**	Fw CTGTGGTTTCCGAGGCTCTT Rv GCATGAGAGGCCAGTGTAGG	60 60	108

**Table 2:** Demographics and clinicopathological characteristics of the included medulloblastoma patients.

Medulloblastoma Patients	N = 23 (100%)
**Gender**	
Male	16 (69.6%)
Female	7 (30.4%)
**Age**	
Age at diagnosis (median, range)	6 (0.5–15) years
Most common age at diagnosis	1 year (13%)
≤3 years	8 (34.8%)
>3 years	15 (65.2%)
**Molecular group**	
WNT	1 (4.4%)
SHH	14 (60.9%)
G3	5 (21.7%)
G4	3 (13.0%)
**Histological group**	
Classic	13 (56.6%)
Nodular Desmoplastic	5 (21.7%)
Extensive nodularity	5 (21.7%)
**Survival**	
Alive	13 (56.5%)
Dead	10 (43.5%)

## Data Availability

The personal data of patients are not available for ethical reasons.

## Supplementary Materials

The following supporting information can be downloaded at: https://www.mdpi.com/article/10.3390/biomedicines13102461/s1. Figure S1: Alignment coverage track for *EZH2* on the based of short-read sequencing data; Table S1: Demographic and clinicopathological characteristics of patients included in *EZH2* sequencing. File S1: Sequences of assembled transcripts.
