# Supplementary material for: Nanopore Sequencing Reveals Novel Alternative Splice Variants of EZH2 in Pediatric Medulloblastoma
Source: Biomedicines. 2025 Oct 10;13(10):2461. doi: 10.3390/biomedicines13102461 (PMC12561525; doi:10.3390/biomedicines13102461)
Supplement: Supplementary file 1 [file biomedicines-13-02461-s001.zip › supplementary file S1.pdf]

Sequences of assembled transcripts.

The sequences of the seven transcripts were assembled via the StringTie method. The intron retention sequences are indicated in bold, and the junction between exons 10 and 12 is highlighted in yellow.

>EZH2_V1

CAGATAAGGGCACAGCAGAAGAACTAAAGGAAAAATATAAAGAACTCACCGAACAGCAGC
TCCCAGGCGCACTTCCTCCTGAATGTACCCCCAACATAGATGGACCAAATGCTAAATCTGT
TCAGAGAGAGCAAAGCTTACACTCCTTTCATACGCTTTTCTGTAGGCGATGTTTTAAATATGA
CTGCTTCCTACATCGTAAGTGCAATTATTCTTTTCATGCAACACCCAACACTTATAAGCGGAA
GAACACAGAAACAGCTCTAGACAACAAACCTTGTGGACCACAGTGTTACCAGCATTTGGA
GGGAGCAAAGGAGTTTGCTGCTGCTCTCACCGCTGAGCGGATAAAGACCCCACCAAAAC
GTCCAGGAGGCCGCAGAAGAGGACGGCTTCCCAATAACAGTAGCAGGCCCAGCACCC
CCACCATTAATGTGCTGGAATCAAAGGATACAGACAGTGATAGGGAAGCAGGGACTGAAA
CGGGGGGAGAGAACAATGATAAAGAAGAAGAAGAGAAGAAAGATGAAACTTCGAGCTCCT
CTGAAGCAAATTCTCGGTGTCAAACACCAATAAAGATGAAGCCAAATATTGAACCTCCTGA
GAATGTGGAGTGGAGTGGTGCTGAAGCCTCAATGTTTAGAGTCCTCATTGGCACTTACTATG
ACAATTTCTGTGCCATTGCTAGGTTAATTGGGACCAAAACATGTAGACAGTGTATGAGTTTAG
AGTCAAAGAATCTAGCATCATAGCTCCAGCTCCCGCTGAGGATGTGGATACTCCTCCAAG
GAAAAAGAAGAGGAAACACCGGTTGTGGGCTGCACACTGCAGAAAGATACAGCTGAAAA
AGGACGGCTCCTCTAACCATGTTTACAACTATCAACCCTGTGATCATCCACGGCAGCCTT

>EZH2_RetI9

CTTTTCATGCAACACCCAACACTTATAAGCGGAAGAACACAGAAACAGCTCTAGACAACAA
ACCTTGTGGACCACAGTGTTACCAGCATTTG**CCACTCCTACCTAGGAACTAAATGGGTAT
ATATTGCCTGTTGGATTTTGGACAGAAGATTCATTGAATGGCACCTGCAGAAGG**GGAGC
AAAGGAGTTTGCTGCTGCTCTCACCGCTGAGCGGATAAAGACCCCACCAAAACGTCCAG
GAGGCCGCAGAAGAGGACGGCTTCCCAATAACAGTAGCAGGCCCAGCACCCCCACCA
TTAATGTGCTGGAATCAAAGGATACAGACAGTGATAGGGAAGCAGGGACTGAAACGGGGG
GAGAGAACAATGATAAAGAAGAAGAAGAGAAGAAAGATGAAACTTCGAGCTCCTCTGAAG
CAAATTCTCGGTGTCAAACACCAATAAAGATGAAGCCAAATATTGAACCTCCTGAGAATGTG
GAGTGGAGTGGTGCTGAAGCCTCAATGTTTAGAGTCCTCATTGGCACTTACTATGACAATTT
CTGTGCCATTGCTAGGTTAATTGGGACCAAAACATGTAGACAGGTGTATGAGTTTAGAGTCA
AAGAATCTAGCATCATAGCTCCAGCTCCCGCTGAGGATGTGGATACTCCTCCAAGGAAAAA
GAAGAGGAAACACCGGTTGTGGGCTGCACACTGCAGAAAGATACAGCTGAAAAAGGACG
GCTCCTCTAACCATGTTTACAACTATCAACCCTGTGATCATCCAC

>EZH2_RetI8RetI9

GAGGAGGAATGGAGAATACGTTGTGATCATTCAGTAAGAGCCTGAAGGAAAGTTGTATG
AACTTTTCATGCAACACCCAACACTTATAAGCGGAAGAACACAGAAACAGCTCTAGACAAC
AAACCTTGTGGACCACAGTGTTACCAGCATTTG**CCACTCCTACCTAGGAACTAAATGGGT**
ATATATTGCCTGTTGGATTTTGGACAGAAGATTCATTGAATGGCACCTGCAGAAGGAGGG
AGCAAAGGAGTTTGCTGCTGCTCTCACCGCTGAGCGGATAAAGACCCCACCAAAACGTC
CAGGAGGCCGCAGAAGAGGACGGCTTCCCAATAACAGTAGCAGGCCCAGCACCCCCA
CCATTAATGTGCTGGAATCAAAGGATACAGACAGTGATAGGGAAGCAGGGACTGAAACGG
GGGGAGAGAACAATGATAAAGAAGAAGAAGAGAAGAAAGATGAAACTTCGAGCTCCTCTG
AAGCAAATTCTCGGTGTCAAACACCAATAAAGATGAAGCCAAATATTGAACCTCCTGAGAAT
GTGGAGTGGAGTGGTGCTGAAGCCTCAATGTTTAGAGTCCTCATTGGCACTTACTATGACAA
TTTCTGTGCCATTGCTAGGTTAATTGGGACCAAAACATGTAGACAGGTGTATGAGTTTAGAGT
CAAAGAATCTAGCATCATAGCTCCAGCTCCCGCTGAGGATGTGGATACTCCTCCAAGGAA
AAAGAAGAGGAAACACCGGTTGTGGGCTGCACACTGCAGAAAGATACAGCTGAAAAAGG
ACGGCTCCTCTAACCATGTTTACAACTATCAACCCTGTGATCATCCACG

>EZH2_RetI9OmE11

CTTTTCATGCAACACCCAACACTTATAAGCGGAAGAACACAGAAACAGCTCTAGACAACAA
ACCTTGTGGACCACAGTGTTACCAGCATTTG**CCACTCCTACCTAGGAACTAAATGGGTAT**
ATATTGCCTGTTGGATTTTGGACAGAAGATTCATTGAATGGCACCTGCAGAAGGAGGGA
GCAAAGGAGTTTGCTGCTGCTCTCACCGCTGAGCGGATAAAGACCCCACCAAAACGTCC
AGGAGGCCGCAGAAGAGGACGGCTTCCCAATAACAGTAGCAGGCCCAGCACCCCCAC
CATTAATGTGCTGGAATCAAAGGATACAGACAGTGATAGGGAAGCAGGGACTGAAACGGG
GGGAGAGAACAATGATAAAGAAGAAGAAGAGAAGAAAGATGAAACTTCGAGCTCCTCTGG
TGTATGAGTTTAGAGTCAAAGAATCTAGCATCATAGCTCCAGCTCCCGCTGAGGATGTGGAT
ACTCCTCCAAGGAAAAAGAAGAGGAAACACCGGTTGTGGGCTGCACACTGCAGAAAGAT
ACAGCTGAAAAAGGACGGCTCCTCTAACCATGTTTACAACTATCAACCCTGTGATCATCCA
CGGCAGCCTT

>EZH2_RetI8OmpE11

GAGGAGGAATGGAGAATACGTTGTGATCATTCAGTAAGAGCCTGAAGGAAAGTTGTATG
AACTTTTCATGCAACACCCAACACTTATAAGCGGAAGAACACAGAAACAGCTCTAGACAAC
AAACCTTGTGGACCACAGTGTTACCAGCATTTGGAGGGAGCAAAGGAGTTTGCTGCTGCT

CTCACCGCTGAGCGGATAAAGACCCCACCAAAACGTCCAGGAGGCCGCAGAAGAGGA
CGGCTTCCCAATAACAGTAGCAGGCCCAGCACCCCCACCATTAATGTGCTGGAATCAAA
GGATACAGACAGTGATAGGGAAGCAGGGACTGAAACGGGGGGAGAGAACAATGATAAAG
AAGAAGAAGAGAAGAAAGATGAAACTTCGAGCTCCTCTGTAATTGGGACCAAAACATGTAG
ACAGGTGTATGAGTTTAGAGTCAAAGAATCTAGCATCATAGCTCCAGCTCCCGCTGAGGAT
GTGGATACTCCTCCAAGGAAAAAGAAGAGGAAACACCGGTTGTGGGCTGCACACTGCAG
AAAGATACAGCTGAAAAAGGACGGCTCCTCTAACCATGTTTACAACTATCAACCCTGTGATC
ATCCACG

>EZH2_RetI8

GAGGAGGAATGGAGAATACGTTGTGATCATTCAGTAAGAGCCTGAAGGAAAGTTGTATG
AACTTTTCATGCAACACCCAACACTTATAAGCGGAAGAACACAGAAACAGCTCTAGACAAC
AAACCTTGTGGACCACAGTGTTACCAGCATTTGGAGGGAGCAAAGGAGTTTGCTGCTGCT
CTCACCGCTGAGCGGATAAAGACCCCACCAAAACGTCCAGGAGGCCGCAGAAGAGGA
CGGCTTCCCAATAACAGTAGCAGGCCCAGCACCCCCACCATTAATGTGCTGGAATCAAA
GGATACAGACAGTGATAGGGAAGCAGGGACTGAAACGGGGGGAGAGAACAATGATAAAG
AAGAAGAAGAGAAGAAAGATGAAACTTCGAGCTCCTCTGAAGCAAATTCTCGGTGTCAAAC
ACCAATAAAGATGAAGCCAAATATTGAACCTCCTGAGAATGTGGAGTGGAGTGGTGCTGAA
GCCTCAATGTTTAGAGTCCTCATTGGCACTTACTATGACAATTTCTGTGCCATTGCTAGGTTA
ATTGGGACCAAAACATGTAGACAGGTGTATGAGTTTAGAGTCAAAGAATCTAGCATCATAGC
TCCAGCTCCCGCTGAGGATGTGGATACTCCTCCAAGGAAAAAGAAGAGGAAACACCGGT
TGTGGGCTGCACACTGCAGAAAGATACAGCTGAAAAAGGACGGCTCCTCTAACCATGTTT
ACAACTATCAACCCTGTGATCATCCACG

>EZH2_RetI8OmE11

GAGGAGGAATGGAGAATACGTTGTGATCATTCAGTAAGAGCCTGAAGGAAAGTTGTATG
AACTTTTCATGCAACACCCAACACTTATAAGCGGAAGAACACAGAAACAGCTCTAGACAAC
AAACCTTGTGGACCACAGTGTTACCAGCATTTGGAGGGAGCAAAGGAGTTTGCTGCTGCT
CTCACCGCTGAGCGGATAAAGACCCCACCAAAACGTCCAGGAGGCCGCAGAAGAGGA
CGGCTTCCCAATAACAGTAGCAGGCCCAGCACCCCCACCATTAATGTGCTGGAATCAAA
GGATACAGACAGTGATAGGGAAGCAGGGACTGAAACGGGGGGAGAGAACAATGATAAAG
AAGAAGAAGAGAAGAAAGATGAAACTTCGAGCTCCTCTGGTGTATGAGTTTAGAGTCAAAG
AATCTAGCATCATAGCTCCAGCTCCCGCTGAGGATGTGGATACTCCTCCAAGGAAAAAGAA
GAGGAAACACCGGTTGTGGGCTGCACACTGCAGAAAGATACAGCTGAAAAAGGACGGCT
CCTCTAACCATGTTTACAACTATCAACCCTGTGATCATCCACG

Complete sequence of EZH2_RetI9 obtained from bioinformatic analysis of RNA-seq data (Illumina) from patients included in the GSE243682 dataset. The intron 9 retention sequence is indicated in bold.>EZH2_RetI9

AGCGATGGCGATTGGGCTGCCGCGTTTGGCGCTCGGTCCGGTCGCGTCCGACACCCG
GTGGGACTCAGAAGGCAGTGGAGCCCCGGCGGCGGCGGCGGCGGCGCGCGGGGG
CGACGCGCGGGAACAACGCGAGTCGGCGCGCGGGACGAAGAATAATCATGGGCCAGA
CTGGGAAGAAATCTGAGAAGGGACCAGTTTGTTGGCGGAAGCGTGTAAAATCAGAGTACAT
GCGACTGAGACAGCTCAAGAGGTTCAGACGAGCTGATGAAGTAAAGAGTATGTTTAGTTCC
AATCGTCAGAAAATTTTGGAAAGAACGGAAATCTTAAACCAAGAATGGAAACAGCGAAGGA
TACAGCCTGTGCACATCCTGACTTCTGTGAGCTCATTGCGCGGGACTAGGGAGTGTTCGG
TGACCAGTGACTTGGATTTTCCAACACAAGTCATCCCATTAAAGACTCTGAATGCAGTTGCT
TCAGTACCCATAATGTATTCTTGGTCTCCCCTACAGCAGAATTTTATGGTGGAAGATGAAACT
GTTTTACATAACATTCCTTATATGGGAGATGAAGTTTTAGATCAGGATGGTACTTTCATTGAAGA
ACTAATAAAAAATTATGATGGGAAAGTACACGGGGATAGAGAATGTGGGTTTATAAATGATGA
AATTTTTGTGGAGTTGGTGAATGCCCTTGGTCAATATAATGATGATGACGATGATGATGATGGA
GACGATCCTGAAGAAAGAGAAGAAAAGCAGAAAGATCTGGAGGATCACCGAGATGATAAA
GAAAGCCGCCCACCTCGGAAATTTCCTTCTGATAAAATTTTTGAAGCCATTTCCTCAATGTTT
CCAGATAAGGGCACAGCAGAAGAACTAAAGGAAAAATATAAAGAACTCACCGAACAGCAG
CTCCCAGGCGCACTTCCTCCTGAATGTACCCCCAACATAGATGGACCAAATGCTAAATCT
GTTCAGAGAGAGCAAAGCTTACACTCCTTTCATACGCTTTTCTGTAGGCGATGTTTTAAATAT
GACTGCTTCCTACATCCTTTTCATGCAACACCCAACACTTATAAGCGGAAGAACACAGAAA
CAGCTCTAGACAACAAACCTTGTGGACCACAGTGTTACCAGCATTTG**CCACTCCTACCTA**
GGAACTAAATGGGTATATATTGCCTGTTGGATTTTGGACAGAAGATTCATTGAATGGCAC
CTGCAGAAGGAGGGAGCAAAGGAGTTTGCTGCTGCTCTCACCGCTGAGCGGATAAAGAC
CCCACCAAAACGTCCAGGAGGCCGCAGAAGAGGACGGCTTCCCAATAACAGTAGCAGG
CCCAGCACCCCCACCATTAATGTGCTGGAATCAAAGGATACAGACAGTGATAGGGAAGCA
GGGACTGAAACGGGGGGAGAGAACAATGATAAAGAAGAAGAAGAGAAGAAAGATGAAAC
TTCGAGCTCCTCTGAAGCAAATTCTCGGTGTCAAACACCAATAAAGATGAAGCCAAATATTG
AACCTCCTGAGAATGTGGAGTGGAGTGGTGCTGAAGCCTCAATGTTTAGAGTCCTCATTGG
CACTTACTATGACAATTTCTGTGCCATTGCTAGGTTAATTGGGACCAAAACATGTAGACAGGT
GTATGAGTTTAGAGTCAAAGAATCTAGCATCATAGCTCCAGCTCCCGCTGAGGATGTGGATA
CTCCTCCAAGGAAAAAGAAGAGGAAACACCGGTTGTGGGCTGCACACTGCAGAAAGATA
CAGCTGAAAAAGGACGGCTCCTCTAACCATGTTTACAACTATCAACCCTGTGATCATCCAC
GGCAGCCTTGTGACAGTTCGTGCCCTTGTGTGATAGCACAAAATTTTTGTGAAAAGTTTTGT
CAATGTAGTTCAGAGTGTCAAAACCGCTTTCCGGGATGCCGCTGCAAAGCACAGTGCAAC
ACCAAGCAGTGCCCGTGCTACCTGGCTGTCCGAGAGTGTGACCCTGACCTCTGTCTTACT
TGTGGAGCCGCTGACCATTGGGACAGTAAAAATGTGTCCTGCAAGAACTGCAGTATTCAGC
GGGGCTCCAAAAAGCATCTATTGCTGGCACCATCTGACGTGGCAGGCTGGGGGATTTTTA

TCAAAGATCCTGTGCAGAAAAATGAATTCATCTCAGAATACTGTGGAGAGATTATTTCTCAAG
ATGAAGCTGACAGAAGAGGGAAAGTGTATGATAAATACATGTGCAGCTTTCTGTTCAACTTG
AACAATGATTTTGTGGTGGATGCAACCCGCAAGGGTAACAAAATTCGTTTTGCAAATCATTC
GGTAAATCCAAACTGCTATGCAAAAGTTATGATGGTTAACGGTGATCACAGGATAGGTATTTT
TGCCAAGAGAGCCATCCAGACTGGCGAAGAGCTGTTTTTTGATTACAGATACAGCCAGGC
TGATGCCCTGAAGTATGTCGGCATCGAAAGAGAAATGGAAATCCCTTGACATCTGCTACCT
CCTCCCCCCTCCTCTGAAACAGCTGCCTTAGCTTCAGGAACCTCGAGTACTGTGGGCAAT
TTAGAAAAAGAACATGCAGTTTGAAATTCTGAATTTGCAAAGTACTGTAAGAATAATTTATAGTA
ATGAGTTTAAAAATCAACTTTTTATTGCCTTCTCACCAGCTGCAAAGTGTTTTGTACCAGTGAA
TTTTTGCAATAATGCAGTATGGTAC
