# Supplementary material for: Nanopore Sequencing Reveals Novel Alternative Splice Variants of EZH2 in Pediatric Medulloblastoma
Source: Biomedicines. 2025 Oct 10;13(10):2461. doi: 10.3390/biomedicines13102461 (PMC12561525; doi:10.3390/biomedicines13102461)
Supplement: Supplementary file 1 [file biomedicines-13-02461-s001.zip › supplementary table S1.pdf]

Supplementary Table S1. Demographic and clinicopathological characteristics of patients included in EZH2 sequencing.

**Patient**	**MB1**	**MB3**	**MB4**	**MB5**	**MB7**	**MB8**	**MB11**	**MB14**
**Gender**	Male	Male	Male	Male	Female	Male	Male	Male
**Age (years)**	0.5	14	15	6	8	4	11	14
**Histological group**	EN	ND	Classic	Classic	Classic	Classic	Classic	Classic
**Molecular group**	SHH	SHH	G4	G3	G3	G3	G4	G4
**Survival status**	Deceased	Alive	Deceased	Alive	Alive	Alive	Alive	Deceased

EN: Extensive Nodularity; ND: Nodular Desmoplastic; G3: Group 3; G4: Group 4.
